# Mechanical injury accentuates lipid deposition in ApoE^–/–^ mice and advance aortic valve stenosis: A novel modified aortic valve stenosis model

**DOI:** 10.3389/fcvm.2023.1119746

**Published:** 2023-02-02

**Authors:** Dezhong Wen, Li Hu, Jianggui Shan, Hengyuan Zhang, Liuhua Hu, Ancai Yuan, Jun Pu, Song Xue

**Affiliations:** ^1^Department of Cardiovascular Surgery, State Key Laboratory for Oncogenes and Related Genes, Renji Hospital, Shanghai Jiao Tong University School of Medicine, Shanghai, China; ^2^Department of Cardiology, Key Laboratory of Coronary Heart Disease, Shanghai Municipal Education Commission, State Key Laboratory for Oncogenes and Related Genes, Renji Hospital, Shanghai Jiao Tong University School of Medicine, Shanghai, China

**Keywords:** aortic valve stenosis, wire injury, hyperlipidemia, calcification, animal model

## Abstract

**Background:**

Current mouse models still have limitations in studying aortic valve stenosis (AVS). A suitable animal model bearing a close resemblance to the pathophysiological processes of humans needs to be developed. Here, we combined two risk factors to create a mouse model that mimics the pathological features of human AVS.

**Methods and results:**

We combined WI and hyperlipidemia in ApoE^–/–^ mice to explore the synergistic effect on the stenosis of the aortic valve. Transthoracic echocardiography revealed progressively increased peak velocity with age in ApoE^–/–^ mice to velocities above C57 mice when fed a high-fat diet after wire injury. Moreover, ApoE^–/–^ mice demonstrated lower cusp separation and lower aortic valve area after 8 weeks vs. C57 mice. Gross morphology and MRI showed advanced thickening, sclerosis aortic valve, narrowing of the orifice area, and micro-CT showed obvious calcification in the aortic valves in the hyperlipidemia group after wire injury. Histopathology studies showed thickening and fibrosis of aortic valve leaflets in the hyperlipidemia group after wire injury. Notably, lipid deposition was observed in ApoE^–/–^ mice 8 weeks after wire injury, accompanied by overexpressed apoB and apoA proteins. After wire injury, the hyperlipidemia group exhibited augmented inflammation, ROS production, and apoptosis in the leaflets. Moreover, the combination group exhibited advanced fibro-calcific aortic valves after wire injury.

**Conclusion:**

Overall, we present the synergistic effect of wire injury and hyperlipidemia on lipoproteins deposition in the development of AVS in ApoE^–/–^ mice, this model bear close resemblance to human AVS pathology.

## 1. Introduction

Aortic valve stenosis (AVS) is the most prevalent heart-valve disease worldwide, and its incidence is increasing with an increasingly elderly population (1, 2). Accumulated evidence suggests that AVS and atherosclerosis share common features including clinical risk factors (advanced age, hypertension, hyperlipidemia, smoking, and diabetes) and histopathological features (endothelial dysfunction, lipid infiltration, inflammation, matrix remodeling, and calcification) (3, 4). Nevertheless, significant differences in the clinical manifestations and therapeutic outcomes differentiate AVS from atherosclerosis. Interestingly, only 40% of patients with AVS have accompanying atherosclerosis, and only a subset of patients with atherosclerosis develop AVS (5). Moreover, inhibitors of HMG-CoA reductase, which were designed to prevent atherosclerosis, failed to retard AVS progression in humans (6, 7). To date, no effective pharmacologic intervention is available for AVS. This unmet medical need is partly attributed to the limited knowledge of the pathophysiological mechanisms of AVS.

Thus, a suitable animal model that closely replicates the pathophysiological processes of AVS is of great importance for research. Considering the advantages of mice over other animals, numerous murine models of AVS have been developed in the last decade. The most commonly used models are the hypercholesterolemic or hyperlipidemic mouse models, which share some features of human AVS, including valve thickening, macrophage accumulation, superoxide production, myofibroblast and osteoblast activation, and mineralization (8, 9). However, almost half a year is required to generate AVS, and few mice develop significant stenosis (4, 10). Systemic inflammation induced by lipopolysaccharides (LPS) was reported to induce AVS (11, 12); however, this method is not widely adopted because the successful development of an AVS model has not been verified. Recently, Honda et al. used a guidewire to induce a novel mouse model of AVS with significant hemodynamic stenosis as early as 4 weeks, based on the response-to-tissue-injury theory (10). However, these mice were free of hyperlipidemia, while the majority of explanted human diseased valves were found to have lipid and lipoprotein deposition in the leaflets (13). However, considerable evidence highlighted the central role of lipids in the progression of AVS (14–16). Therefore, these models are of limited use in clarify the specific mechanisms in AVS development. Moreover, most mouse models emphasize a single risk factor for the development of AVS, whereas AVS is driven by multiple risk factors in humans.

Therefore, in this study, we induced wire injury (WI) to the leaflet in hyperlipidemic ApoE^–/–^ knockout mice to evaluate the synergistic effect of these two risk factors on lipoprotein deposition and the development of AVS. This simple murine model, in which severe AVS develops rapidly, replicates the pathophysiological processes of human AVS.

## 2. Materials and methods

### 2.1. Animals and aortic valve stenosis

The animal studies were approved by the Animal Ethics Committee of Ren Ji Hospital of Shanghai Jiao Tong University School of Medicine and carried out in accordance with the NIH guidelines (Guide for the care and use of laboratory animals). Adult male C57BL/6J mice and male ApoE^–/–^ mice (C57BL/6J background) aged 6–8 weeks were purchased from GemPharmatech (Jiangsu, China), housed in a pathogen-free, temperature-controlled environment under a 12:12 h light-dark cycle.

Aortic-valve injury was induced as previously described with minor modification (10, 17). Briefly, the mice were anesthetized by intraperitoneal injection of a solution comprising 150 mg/kg ketamine and 16 mg/kg xylazine, and a spring wire was introduced into the carotid artery. The wire was passed over the aortic valve under echocardiography guidance and rotated over the valve 110 times. Additionally, the leaflets were scratched with the body of the wire 30 times. The carotid artery was ligated before closing the skin incision. Sham surgery was performed in the same way without advancing the wire across the aortic valve into the left ventricle. After surgery, C57BL/6J mice received normal chow or a Western diet (TD88137; Harlan Teklad), ApoE^–/–^ mice fed with a Western diet (TD88137; Harlan Teklad).

### 2.2. Echocardiography

Transthoracic echocardiography was performed under 2.5% isoflurane anesthesia. The images were acquired by Vevo 2100 Imaging system (Visualsonics, Toronto, Canada). Left ventricular ejection fraction (EF), fractional shortening (FS), and ventricular volumes were measured in parasternal long-axis view using the LV-Trace function. Aortic valve peak velocity was measured in the suprasternal view with a pulse-wave-Doppler using angle correction between 45° and 55°. Aortic valve area (AVA) was calculated by the continuity equation. *M*-Mode was used for cusp-separation evaluation in the left-parasternal-long axis view using the inner-edge to inner-edge convention to assess distance. The average of three consecutive cardiac cycles was used for each measurement.

### 2.3. Micro-computed tomography

CT scanning of mice was acquired by a micro-CT scanner (Inviscan, IRIS, France) 16 weeks after wire injury. After induction of anesthesia (1 L/min airflow rate and ≈1.5% isoflurane), the mice were transferred into the attached CT scanner and imaged. Images were analyzed using the Inviscan research workplace software.

### 2.4. Magnetic resonance imaging

Imaging was performed using a 7 T Bruker Pharmascan (Bruker BioSpin, Ettlingen, Germany) as described previously (18). During imaging, the animals were anesthetized with 1−2% isoflurane. Electrocardiography and respiratory signals were measured using an MRI-compatible monitoring unit (SA Instruments, Inc., NY, USA). Aortic valve function was examined by imaging the cross-section of the aorta at the level of the valve using a cine sequence with a slice thickness of 0.6 mm. Other parameters were as follows: TR = 8 ms, TE = 2 ms, FOV = 1.5 × 3 cm^2^, matrix size 192 × 192 resulting in plane pixel size of 78 × 156 μm^2^. The average of three measurements was used for analysis. Three parallel slices were collected, and the slice showing all three cusps the most clearly was selected for analysis. Images were analyzed by Circle Cardiovascular Imaging software package (5.5.6.1).

### 2.5. Histopathological assessment

#### 2.5.1. Anatomic features of the aortic valve

Hearts explanted from mice were perfused with saline to remove blood, followed by perfusion with 4% paraformaldehyde (PFA) for 30 min to fix the aortic valves. This perfusion keeps the aortic valves open irrespective of the phase of the heart at the time of cardiac arrest. Aortic vessels were trimmed around the valves, images were obtained by a stereoscopic microscopy (SMZ745, Nikon, Japan).

#### 2.5.2. Morphological characterization

Mice were sacrificed with a lethal dose of pentobarbital sodium (100 mg/kg), and hearts were collected, fixed in 4% paraformaldehyde (PFA), and embedded in paraffin or Tissue-Tek OCT compound (Sakura). Cross-sections of the aortic valves (5 μm thick) were stained with hematoxylin-eosin (HE). Changes in the collagen content after WI were detected by Masson’s Trichrome staining. Movat’s Pentachrome staining was used to visualize proteoglycans in the aortic valve as described previously (19). For Oil Red O staining, frozen sections were stained with Oil Red O and hematoxylin. Tissue calcification was measured using Von kossa staining. Images were obtained using a Nano Zoomer S360 scanner (Hamamatsu Photonics, Japan). Leaflet thickness was measured using NDP view 2 software (version 2.7.43; Hamamatsu Photonics, Japan) as previously reported (20, 21). The relative areas of fibrosis and calcification after Oil Red O and von Kossa staining, respectively, were analyzed by Image J software (version 1.53c; National Institutes of Health).

#### 2.5.3. Immunohistochemistry/immunofluorescence staining

For immunohistochemistry, sections were incubated with antibodies against apoB (1:50 Proteintech), apoA (1:50 Proteintech), F4/80 (1:50, ab111101, Abcam). For immunofluorescence staining, sections were blocked with 5% bovine serum albumin in phosphate-buffered saline (PBS) for 1 h. Incubations with primary antibodies was performed overnight at 4°C at the following dilutions: Runx2 (1:50, AF2593, Beyotime), α−smooth muscle actin (α−SMA, 1:250, ab7817, Abcam), VCAM−1 (1:25, MA5−11447, Invitrogen), MCP−1 (1:200, MA5−17040, Invitrogen), Collagen I (1:200, GB11022−3, Servicebio), Collagen III (1:200, ab7778, Abcam), and CD31 (1:50, ab28364, Abcam). After primary antibody incubation, the sections were washed in PBS and incubated with Alexa 488 anti-rabbit (1:500, A21206, Invitrogen) and Alexa 555 anti-mouse (1:500, A−31570, Invitrogen) secondary antibodies for 1 h at room temperature. Subsequently, the slides were washed and counterstained with 4′,6−diamidino-2-phenylindole (DAPI, 1:10,000, Life Technologies) for the identification of nuclei. Images were obtained by a fluorescence microscopy (DMI2500, Leica, Germany). Fluorescence intensity corrected by background intensity was quantified using ImageJ software (version 1.53c; National Institutes of Health) as described previously (22).

#### 2.5.4. Superoxide detection and TUNEL staining

Dihydroethidium staining was used to detect reactive oxygen species (ROS) after surgery. Briefly, unfixed frozen sections (5 μm) were incubated with dihydroethidium (DHE, 10 μmol/L) at 37°C for 30 min, followed by 5−min wash in PBS. An *in situ* cell death detection kit was used to detect aortic valve cell apoptosis during stenosis, following the manufacturer’s protocol (TUNEL Apoptosis Detection Kit, FITC, Yeasen). The nuclei were counterstained with DAPI. Then, the images were obtained with a fluorescence microscopy (DMI2500, Leica, Germany). Images were analyzed using Image J software.

### 2.6. Blood biochemical analysis

Animals were sacrificed and blood was collected for further analysis. Detection of serum total cholesterol (TC), low-density lipoproteins (LDL), high-density lipoproteins (HDL), and triglyceride (TG) levels were performed using indicated kits (Biosino Biotechnology Company Ltd., China).

### 2.7. Statistical analysis

All values are presented as mean ± SEM and analyzed using the Student’s *t*-test for two groups were compared. Where only chow diet, WI, or genotype was described, a one-way ANOVA was used followed by Tukey–Kramer *post-hoc* test. For analysis of multiple genotypes and diets, two-way ANOVA followed by Tukey–Kramer *post-hoc* test was performed. Statistical analyses were performed with SPSS 13.0 statistical software (SPSS Inc., Chicago, USA). *P* < 0.05 was considered to be statistically significant.

## 3. Results

### 3.1. Combined mechanical injury and hyperlipidemia aggravated calcific aortic valve stenosis

**FIGURE 1:**
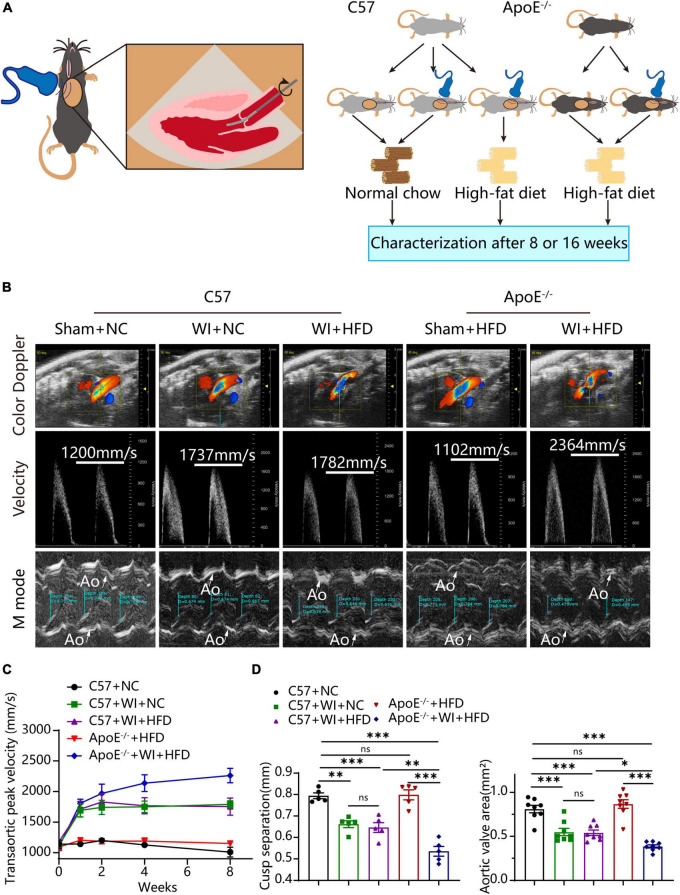
Combined mechanical injury and hyperlipidemia caused aortic valve stenosis. **(A)** Schematic overview of the experimental strategy. C57 or ApoE^–/–^ mice treated with wire injury and received normal chow (NC) or high-fat diet (HFD) for 8 or 16 weeks. **(B)** Representative color doppler, transaortic peak velocity and cusp separation 8 weeks after wire injury. White arrows indicated the aorta (Ao). **(C)** Time course of aortic valve velocity evaluated by echocardiography at 0, 1, 2, 4, 8 weeks after wire injury (*n* = 8 per group). **(D)** Representative cusp separation (*n* = 5 per group) and aortic valve area evaluated 8 weeks after wire injury (*n* = 8 per group). Results are mean ± SEM. Adjusted *P* values were provided in case of multiple groups. ns. indicates non-significant *P* value. *P < 0.05, **P < 0.01, and ***P < 0.001.

**FIGURE 2:**
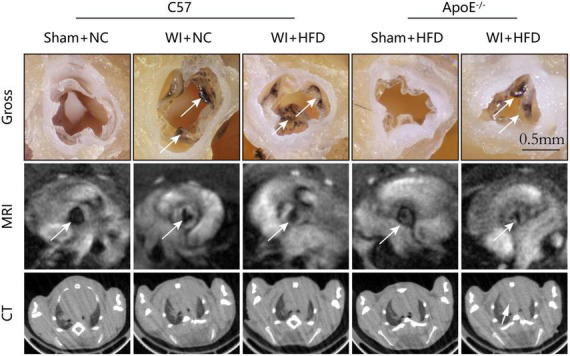
Aortic valve morphology and calcification examined by MRI and Micro-CT. Representative gross morphology (**top**, scale bar, 0.5 mm), MRI images of the aortic valve **(middle)**, and CT images of aortic calcification **(bottom)**. The sclerotic leaflets were marked by the white arrow after wire injury in the **top panel**, the white arrows point toward the aortic valve orifice in the **middle panel**, and the white arrow indicates the aortic calcification in the **bottom panel**.

To develop a mouse model with rapid severe aortic valve stenosis and lipid deposition, we combined mechanical injury and hyperlipidemia in ApoE^–/–^ knockout mice. After surgery, C57 and ApoE^–/–^ mice were fed with NC or high-fat diet (HFD) for 8 or 16 weeks (Figure 1A). Next, we performed a time-course analysis of transaortic peak velocity after WI for up to 8 weeks, and the aortic valve area and aortic valve cusp separation were evaluated at 8 weeks (Figures 1B–D). The mice that received WI both had progressively increased peak velocity with age and velocities above those in the C57 + NC and ApoE^–/–^ + HFD groups (>1600 mm/s) (Figure 1C). Compared with C57 + WI + HFD mice, ApoE^–/–^ + WI + HFD mice exhibited a significant increase in peak velocity and a significant decrease in aortic valve cusp separation and aortic valve area (Figures 1C, D). However, there was no significant difference in peak velocity, aortic valve cusp separation, and aortic valve area between C57 + WI + NC and C57 + WI + HFD mice (Figures 1C, D). Feeding ApoE^–/–^ mice with HFD for 20 weeks is a classic method to develop AVS. However, in our study, ApoE^–/–^ mice did not develop obvious AVS after 8 weeks of HFD. The echocardiographic assessment showed no significant difference in systemic hemodynamic parameters between the groups at the end of the study (Table 1). Stereoscopic microscopy showed obvious thickening of the leaflets and a significant reduction in the aortic valve opening orifice in ApoE^–/–^ + WI + HFD mice compared with C57 + WI + HFD mice (Figure 2). The results of the aortic valve orifice area observed by magnetic resonance imaging were in line with results observed by stereoscopic microscopy. This synergistic effect was further documented using micro-CT 16 weeks after WI, confirming that advanced deposition of calcium hydroxyapatite occurred in ApoE^–/–^ + WI + HFD group (Figure 2).

**TABLE 1:** Echocardiographic parameters in different groups of mice.

	C57			ApoE^–/–^	
Parameters	NC (*n* = 8)	WI + NC (*n* = 8)	WI + HFD (*n* = 8)	HFD (*n* = 8)	WI + HFD (*n* = 8)
BW before (g)	20.71 ± 0.2943	21.56 ± 0.1614	20.86 ± 0.1592	19.50 ± 0.2570	20.19 ± 0.5252
BW after (g)	26.90 ± 0.3808	27.39 ± 0.6572	30.30 ± 0.8842	26.45 ± 0.5892	28.78 ± 1.034
Heart rate (bpm.)	481.9 ± 11.55	499.7 ± 5.173	508.6 ± 6.495	509.0 ± 5.015	509.2 ± 6.308
CO (ml/min)	16.70 ± 1.581	20.39 ± 1.132	19.43 ± 1.751	18.09 ± 1.137	19.25 ± 1.347
SV (ml)	34.71 ± 3.167	40.81 ± 2.374	38.12 ± 3.279	35.50 ± 2.116	38.33 ± 2.622
IVSd (mm)	0.7603 ± 0.04889	0.8153 ± 0.03333	0.7750 ± 0.03417	0.7870 ± 0.06187	0.8323 ± 0.03092
PWd (mm)	0.6346 ± 0.04637	0.5645 ± 0.03125	0.5553 ± 0.02830	0.5803 ± 0.03770	0.6162 ± 0.03277
LVEDd (mm)	3.524 ± 0.09762	3.703 ± 0.1638	3.831 ± 0.1431	3.676 ± 0.09880	3.734 ± 0.1219
EF (%)	65.96 ± 3.742	71.13 ± 4.002	59.91 ± 3.067	62.43 ± 3.604	70.64 ± 1.270
FS (%)	36.14 ± 2.792	40.83 ± 3.599	31.67 ± 2.117	33.47 ± 2.505	39.38 ± 1.103
Transaortic peak velocity (mm/s)	1010 ± 80.15	1790 ± 58.66***	1753 ± 139.8	1152 ± 29.14	2261 ± 117.6##‡
AVA (mm^2^)	0.8100 ± 0.04265	0.5489 ± 0.04154***	0.5406 ± 0.03140	0.8679 ± 0.04904	0.3855 ± 0.01797#‡

Data are presented as mean ± SEM. NC, normal chow; HFD, high-fat diet; WI, wire injury; AVA, aortic valve area; BW, body weight; FS, function of shortening; IVSd, intraventricular septal diameter; LVEDd, left ventricular end-diastolic diameter; PWd, posterior wall diameter.

***P < 0.001 vs. C57 + NC, ^#^*P* < 0.05 vs. C57 + WI + HFD, ^##^*P* < 0.01 vs. C57 + WI + HFD, ^‡^*P* < 0.001 vs. ApoE^–/–^+ HFD.

### 3.2. Combined mechanical injury and hyperlipidemia induced advanced aortic valve pathological remodeling

**FIGURE 3:**
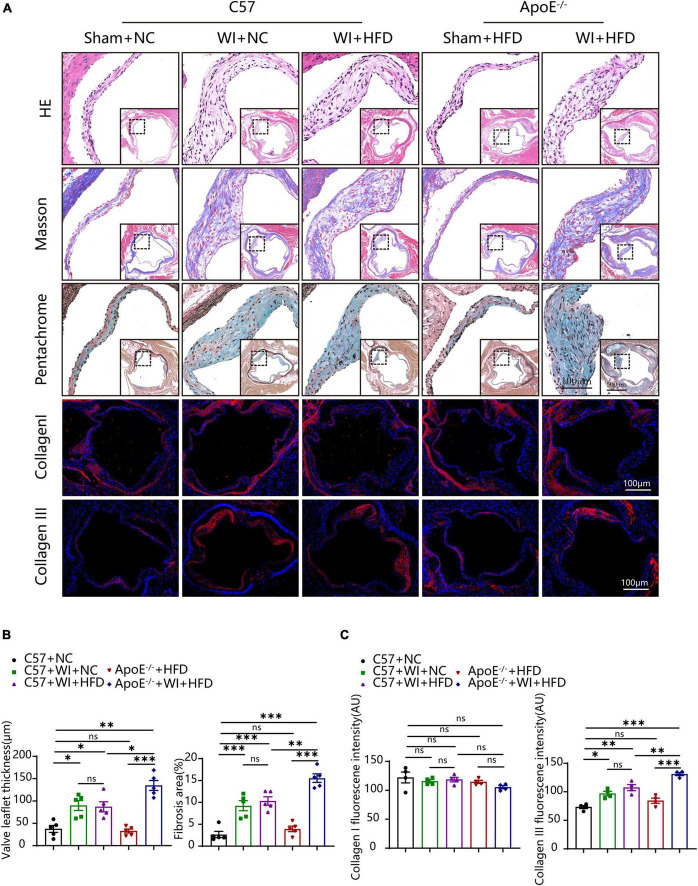
Combined mechanical injury and hyperlipidemia induced advanced aortic valve pathological changes. **(A)** Pathological changes of murine aortic valve 8 weeks after wire injury in the indicated groups stained with HE, Masson, Pentachrome (large, scale bar, 100 μm; small, scale bar, 500 μm) and immunofluorescence of Collagen I and Collagen III (scale bar, 100 μm). **(B)** Quantification of the valve leaflet thickness and the fibrosis area of murine aortic valve 8 weeks after wire injury in the indicated groups (*n* = 5 per group). **(C)** Quantification of fluorescent intensity of Collagen I and Collagen III. Results are mean ± SEM. Adjusted *P* values were provided in case of multiple groups. ns. indicates non-significant *P* value. *P < 0.05, **P < 0.01, and ***P < 0.001.

In addition to multimodal imaging measurements to evaluate the stenosis of the aortic valve, histopathological staining was performed to characterize aortic valve remodeling after WI. Morphological analysis supported the synergistic effect that advanced the process of the disease in aortic valve remodeling after the procedure. The average leaflet thickness was significantly increased after WI, and hyperlipidemia further aggravated the thickness of the leaflet (Figures 3A, B). Next, we evaluated the changes in extracellular matrix composition in different groups. In control groups, collagen and glycosaminoglycan (GAG) distribution was restricted to the thin fibrosa layer of the valve, whereas, excessive collagen and GAGs accumulated and extended to the whole valve area in the WI groups (Figure 3A). This trend was further accentuated in the hyperlipidemic group after WI. The fibrosis area in ApoE^–/–^ + WI + HFD mice was significantly larger than that in C57 + WI + HFD mice (Figures 3A, B). Furthermore, we explored the changes in the collagen content in different groups. The proportion of collagen I in the aortic valve was equal in all groups, whereas that of collagen III was significantly higher in the surgery groups; the increase was greater in the hyperlipidemic group after WI. However, there was no significant difference in the average leaflet thickness, fibrosis area, GAGs, and collagen content between the C57 + WI + NC mice and C57 + WI + HFD mice (Figures 3A–C).

### 3.3. Mechanical injury disrupted integrity of the endothelium and advanced fatty and lipoproteins deposition beneath the leaflet endothelium

**FIGURE 4:**
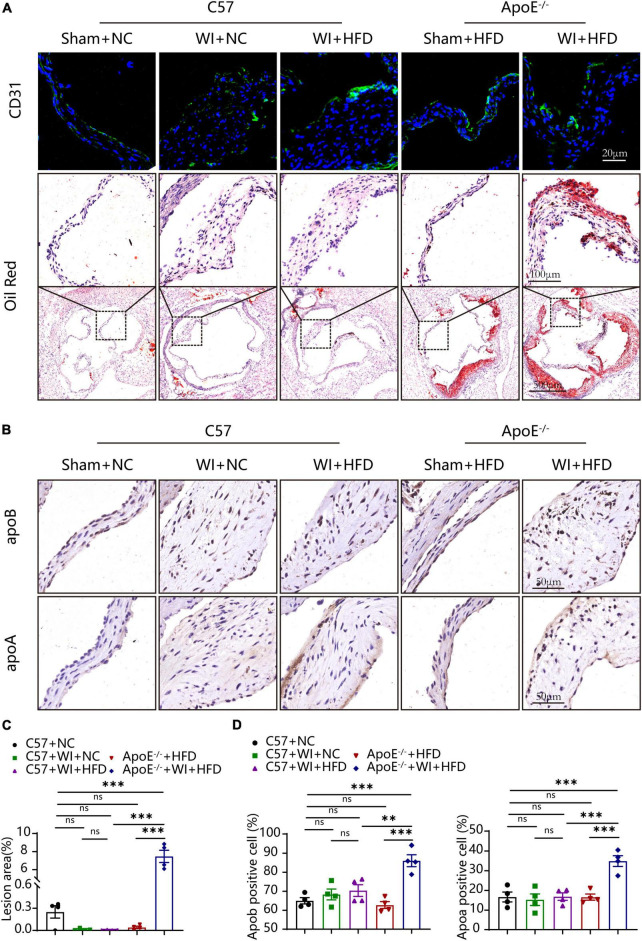
Mechanical injury and hyperlipidemia induced advanced fatty and lipoproteins deposition beneath the leaflets endothelial. **(A)** Representative CD31 immunostaining (green, scale bar, 20 μm) and Oil red O staining (top, scale bar, 100 μm; bottom, scale bar, 500 μm) of aortic valves 8 weeks after wire injury. Valvular lipid deposition was only observed in hypercholesterolemic mice 8 weeks after wire injury. **(B)** Representative images of apoB (scale bar, 50 μm) and apoA (scale bar, 50 μm) lipoproteins in the valvular 8 weeks after wire injury in the indicated groups (*n* = 4 per group). **(C)** Quantification of the valvular lesion area (%) 8 weeks after wire injury in the indicated groups (*n* = 4 per group). **(D)** Quantification of apoB (scale bar, 50 μm) and apoA (scale bar, 50 μm) lipoproteins in the valvular 8 weeks after wire injury in the indicated groups (*n* = 4 per group). Results are mean ± SEM. Adjusted *P* values were provided in case of multiple groups. ns. indicates non-significant *P* value. **P < 0.01 and ***P < 0.001.

The integrity of the endothelial layer plays an important role in modulating valvular homeostasis (23). Damage to the aortic valve endothelium favors the infiltration of inflammatory cells and deposition of lipids and is considered as the first step of AVS (24, 25). In an attempt to explore endothelial damage, we assessed the endothelial integrity based on immunofluorescence of CD31, which revealed a discontinuous of the endothelium after WI (Figure 4A), indicating that WI provided the possibility for lipid deposition and macrophage aggregation. As lipoproteins play a crucial role in driving the progression of AVS (26, 27), we performed Oil Red O staining to verify whether our model is suitable for studying lipid or lipid-derived compounds in the development of AVS. Advanced lipid deposition was observed in the leaflets or aortic sinus in ApoE^–/–^ + WI + HFD mice (Figure 4A), and the leaflet lesion area was approximately 7.474% (Figure 4C). Meanwhile, the levels of TC and low-density lipoprotein (LDL) were 2 times and 14 times higher than those in the C57 + WI + HFD mice, respectively (Table 2). Although C57 + WI + HFD mice showed a slight increase in TC and HDL (Table 2), no lipid deposition was found in the leaflet or aortic sinus. ApoE^–/–^ mice fed on HFD spontaneously develop atheroma plaques. However, in our study, although obvious lipid deposit was observed at the level of the aortic sinus, no lipid deposit was observed in the leaflet of ApoE^–/–^ + HFD mice. Nonetheless, TC and LDL levels were 4 times and 15 times higher, respectively, than those in C57 + NC mice at 8 weeks (Table 2), which indicated that short hyperlipidemia duration may not damage the integrity of the endothelial layer. Moreover, significant increases in apoB and apoA levels were observed in ApoE^–/–^ + WI + HFD mice compared to C57 + WI + HFD mice (Figures 4B, D). Although other groups showed similar trends, the differences were not statistically significant (Figures 4C, D).

**TABLE 2:** Metabolic parameters in different groups of mice.

	C57			ApoE^–/–^	
Parameters	NC (*n* = 5)	WI + NC (*n* = 5)	WI + HFD (*n* = 5)	HFD (*n* = 5)	WI + HFD (*n* = 5)
TC (mmol/l)	3.012 ± 0.09836	2.742 ± 0.05663	4.848 ± 0.3013	13.05 ± 1.298***	10.56 ± 0.4373###
TG (mmol/l)	1.539 ± 0.1522	1.437 ± 0.1702	1.067 ± 0.07682	1.265 ± 0.1327	1.153 ± 0.1257
LDL (mmol/l)	0.422 ± 0.01	0.4074 ± 0.02793	0.4368 ± 0.02895	6.504 ± 1.336***	5.716 ± 0.282###
HDL (mmol/l)	1.827 ± 0.1045	1.629 ± 0.04340	2.941 ± 0.1499‡	2.047 ± 0.1162	1.750 ± 0.1060###

Data are presented as mean ± SEM. TC, total cholesterol; TG, triglyceride; LDL, low density lipoprotein; HDL, high density lipoprotein.

***P < 0.001 vs. C57 + NC, ^###^*P* < 0.001 vs. C57 + WI + HFD, ^‡^*P* < 0.001 vs. C57 + WI + HFD.

In summary, mechanical injury only led to disruption of endothelial integrity, whereas ApoE^–/–^ mice only exhibited hypercholesterolemia rather than fatty and lipoproteins deposition in 8 weeks. However, combining these two stimulating factors resulted in advanced thickening of the aortic valve with atherosclerotic lesions in the leaflet. These results suggest that this is an appropriate model for studying AVS.

### 3.4. Combined mechanical injury and hyperlipidemia augmented advanced superoxide reaction and inflammation

**FIGURE 5:**
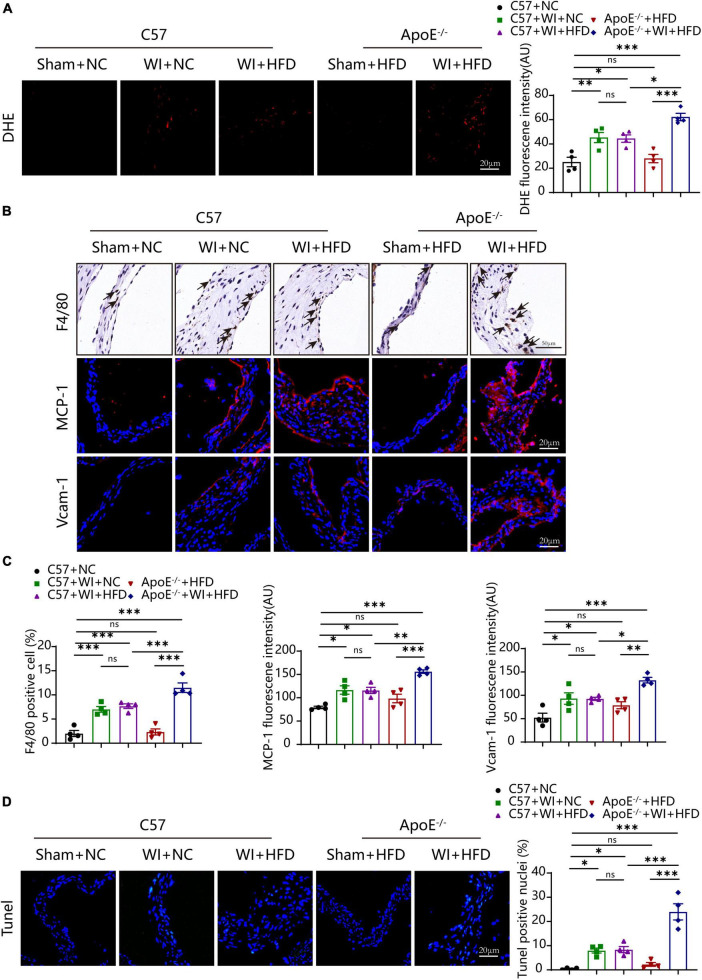
Hyperlipidemia augmented advanced superoxide reaction and inflammation. Hyperlipidemia exacerbated superoxide (DHE, red, scale bar, 20 μm); **(A)** after wire injury. Hyperlipidemia induced increment of macrophage accumulation beneath the leaflets endothelial (scale bar, 50 μm) after wire injury and resulted in a pro-inflammatory valvular with increased expression of the chemokine MCP−1 and Vcam−1 (red, scale bar, 20 μm; **(B)**. Quantitative analysis of macrophage (%), MCP−1, and Vcam−1 **(C)**, *n* = 4 per group. Hyperlipidemia exacerbated apoptosis (Tunel, green, scale bar, 20 μm) after wire injury **(D)**, *n* = 4 per group. Results are mean ± SEM. Adjusted *P* values were provided in case of multiple groups. ns. indicates non-significant *P* value. *P < 0.05, **P < 0.01, and ***P < 0.001.

Lipid oxidation reaction triggers an inflammatory response in the leaflet, and both are the stimulus of calcification (25, 28). We next evaluated whether the combination of WI and hyperlipidemia causes advanced superoxide reaction and inflammation. The oxidative stress level increased after WI; notably, ApoE^–/–^ + WI + HFD mice showed significantly higher oxidative stress as compared with C57 + WI + HFD mice (Figure 5A). In control mice and ApoE^–/–^ + HFD mice, macrophages were rarely detected under the leaflet endothelium of the aortic valve (Figure 5B). However, macrophage infiltration increased after WI, ApoE^–/–^ + WI + HFD mice exhibited a significant increase in macrophage infiltration compared to C57 + WI + HFD mice (Figures 5B, C). As the migratory capacity of the monocytes is stimulated by chemoattractant cytokines and chemokines recruit monocytes to sites of damaged endothelial (29). To this end, we assessed changes of the markers for leukocyte recruitment. Consistent with the observed increase in macrophage infiltration, significantly increased expression of MCP−1 and Vcam−1 was observed in the injured leaflets. In particular, a significantly higher expression of MCP−1 and Vcam−1 was observed in ApoE^–/–^ + WI + HFD mice compared to that in C57 + WI + HFD mice (Figures 5B, C), indicating that combined mechanical injury and hyperlipidemia result in more pro-inflammatory changes. Stimulated by adverse factors, valvular interstitial cells (VICs) undergo apoptosis and release apoptotic bodies that act as nucleation sites for microcalcification (30). Therefore, we further examined the apoptosis in the aortic valve after WI in different groups. Significantly increased Tunel- positive cells were observed in WI groups compared with controls. The percentage of the Tunel-positive cells was markedly increased in ApoE^–/–^ + WI + HFD mice as compared with that in C57 + WI + HFD mice (Figures 5C, D). No significant differences were observed between C57 + WI + NC mice and C57 + WI + HFD mice with regard to macrophage infiltration, MCP−1 and Vcam−1 expression, level of oxidative stress, and apoptosis.

### 3.5. Hyperlipidemia augmented myofibroblast activation and osteogenic reaction

**FIGURE 6:**
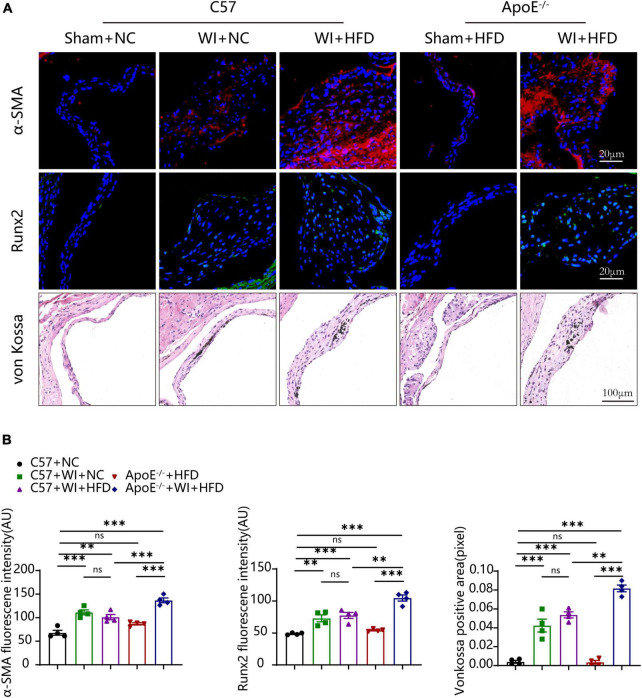
Hyperlipidemia augmented advanced myofibroblast activation and Osteogenic reaction. **(A)** Representative images of α-Smooth muscle actin (α-SMA, red, scale bar, 20 μm), Runx2 (green, scale bar, 20 μm) immunostaining and von Kossa (scale bar, 100 μm) of aortic valves 8 weeks after wire injury and quantification of immunofluorescent data, *n* = 4 per group. Higher expression of α-SMA and Runx2 were observed in hyperlipidemic mice after wire injury. **(B)** Quantitative analysis of α-SMA, Runx2 immunofluorescent, and von Kossa positive area data, *n* = 4 per group. Results are mean ± SEM. Adjusted *P* values were provided in case of multiple groups. ns. indicates non-significant *P* value. **P < 0.01 and ***P < 0.001.

Under normal conditions, VICs are predominantly quiescent fibroblasts, with a small population of smooth muscle cells (31). Under the stimulation of the adverse factors, quiescent VICs differentiate into myofibroblasts (32). Immunofluorescence showed myofibroblast activation as α-smooth muscle actin (α-SMA) fluorescence intensity in wire injury groups was more vigorous than controls (Figure 6A). The fluorescence intensity of α-SMA in ApoE^–/–^ + WI + HFD mice was markedly higher than that in C57 + WI + HFD mice (Figures 6A, B). Calcification is an important hallmark at the propagation phase of the AVS (33). To determine the synergistic effect of the combination of mechanical injury and hyperlipidemia on calcification, the specimen were performed with Runx2 and von Kossa staining, which indicate osteogenic differentiation and mineral deposition, respectively. As revealed, mechanical injury induced higher osteogenic differentiation and mineral deposition as increased expression of Runx2 and von Kossa positive area were observed when compared with controls. When combined with hyperlipidemia, these two factors showed a synergistic effect on calcification, characterized by significantly increased expression of Runx2 and von Kossa positive areas (Figures 6A, B). However, the C57 + WI + NC and C57 + WI + HFD groups showed similar results in the expression of α-SMA, Runx2, and von Kossa positive areas.

## 4. Discussion

Aortic valve stenosis is a multi-etiological disease, and emerging evidence has revealed that it is an active disease involving cellular and molecular pathways (25). Several animal models have been developed to clarify the mechanism of AVS; diet and genetic mouse models and WI mouse models are the most widely used (10, 34). However, both of these two different models have some limitations, diet and genetic mouse models require more than half a year to develop AVS and rarely develop significant stenosis, and the WI model develops rapid significant stenosis but is free of lipid deposition. As hyperlipidemia and mechanical injury are both pathological inducers of AVS, we hypothesized that the combination of these two factors might have a synergistic effect in the development of a rapid stenosis mouse model with significant atherosclerosis. Combining these two factors, we generated a model of rapid hemodynamically significant stenosis accompanied by pronounced lipid deposition. Eight weeks after surgery, the animals showed significantly increased aortic valve velocity, with thickening and sclerosis of the aortic valve. In hyperlipidemic animals, significant adverse aortic-valve remodeling was observed after surgery, including increased collagen and GAGs accumulation. The disruption of the integrity of endothelial and fatty deposition were detected after surgery in hyperlipidemic animals accompanied by increased lipoproteins deposition. Moreover, disruption of the endothelial integrity, fat deposition accompanied by increased lipoprotein deposition, increased oxidative stress, inflammation, and apoptosis were observed after surgery in hyperlipidemic animals. Furthermore, increased myofibroblast activation and osteogenic reactions were observed in hyperlipidemic animals. These observations are concordant with the fact that the development of aortic stenosis is driven by multiple risk factors in humans (14, 32).

Endothelial dysfunction induced by mechanical and (oscillatory) shear stress is regarded as the first step of AVS (24). This theory is reinforced by the predisposition and rapid progression of AVS in patients with bicuspid valves with high mechanical stress (24). Endothelial disruption facilitates lipid deposition, which is a hallmark of the early stage of AVS. To substantiate the synergistic effect of these two factors, the animals were fed NC or HFD after WI. After surgery, all animals showed a progressively increased peak velocity with age, accompanied by decreased cusp separation and aortic valve area, which indicated the success of the surgery. The discontinuity of CD31 immunofluorescence indicated disruption of the integrity of endothelial after WI. Although C57 mice were treated with HFD for 8 weeks after WI, it was insufficient to contribute to the deposition of lipids, as these animals only showed a slight increase in TC and HDL levels. ApoE^–/–^ mice treated with HFD for 8 weeks exhibited severe hypercholesterolemia, but no change in the aortic valve, as these mice usually take more than half a year to develop AVS. However, when combined with WI, ApoE^–/–^ mice exhibited rapidly increased aortic valve velocity and decreased aortic valve area and cusp separation, and the stenosis trend was more severe than that in C57 mice after surgery. Moreover, thickening aortic valves were observed in all surgery groups, and the combined groups showed more thickened and sclerotic leaflets with the adverse remodeling of the extracellular matrix, which indicates both hypercholesterolemia and mechanical injury contribute to AVS progression. Lipid deposition was only observed in the combined groups, indicating that disruption of endothelial integrity facilitated lipid accumulation. Increased expression of apolipoproteins, such as apoA and apoB were observed in the combined groups, which was in line with the overexpression of apoA and apoB in human explants (13).

Previous studies have shown that lipids and lipid-derived compounds strongly induce chronic inflammation and calcification. Increased oxidative stress in the diseased aortic valve promotes the formation of ox-LDLs (oxidized low-density lipoproteins) and oxidized phospholipids (35). Ox-LDLs further trigger pro-inflammatory cytokines expression and consequently recruit immune cells to migrate into lesions (36–38). Lipoprotein(a) and its associated bioactive compounds, including lipoprotein-associated phospholipase A2, oxidized phospholipids, and autotaxin, are overexpressed in human plasma and aortic valve explants. Lipoprotein(a) has been shown to induce VICs to undergo osteogenic differentiation, apoptosis, calcium deposition, and ROS production (27, 39, 40), highlighting a link between lipid metabolism and AVS. In our study, increased inflammation, ROS production, apoptosis, and osteogenic differentiation were observed in the combined groups, demonstrating that this mouse model nicely mimicked most hallmarks of the human aortic-valve disease.

Although the mouse model used in this study nicely replicates the pathophysiological processes of human AVS, it had several limitations. First, pathological changes occurred at the level of the free edge of the leaflets after WI whereas that pathological changes mainly occurred at the level of the aortic side of the leaflets in humans. Second, the animals did not show impaired cardiac function after WI, probably due to insufficient afterload in our model. At 8 weeks, the aortic valve velocity in our study was around 2,000 mm/s, whereas the maximum aortic valve velocity reported by Honda et al. was 3,830 mm/s (10). Second, we did not clarify the specific mechanisms that may contribute to AVS in this model; therefore, further in-depth studies are warranted.

In conclusion, this study demonstrated a synergistic effect of WI and hyperlipidemia on the development of AVS in ApoE^–/–^ mice. We successfully modified the WI model, and the methods adopted in this study were simple, fast, and highly efficient. This model bear close resemblance to human AVS pathology with rapid sclerosis aortic valve will be helpful in expanding our knowledge of the mechanisms of AVS and testing effective pharmacological therapies.

## Data availability statement

The original contributions presented in this study are included in the article/supplementary material, further inquiries can be directed to the corresponding authors.

## Ethics statement

This animal study was reviewed and approved by Animal Ethics Committee of Renji Hospital of Shanghai Jiao Tong University School of Medicine.

## Author contributions

SX and JP designed the study and revised the manuscript. DW and LiH conducted the research and wrote the manuscript. JS, HZ, LiuH, and AY participated in analyzing the data. All authors contributed to the article and approved the submitted version.
